# *SAMHD1* Gene Mutations Are Associated with Cerebral Large-Artery Atherosclerosis

**DOI:** 10.1155/2015/739586

**Published:** 2015-10-04

**Authors:** Wei Li, Baozhong Xin, Junpeng Yan, Ying Wu, Bo Hu, Liping Liu, Yilong Wang, Jinwoo Ahn, Jacek Skowronski, Zaiqiang Zhang, Yongjun Wang, Heng Wang

**Affiliations:** ^1^Department of Neurology, Beijing Tiantan Hospital, Capital Medical University, Beijing 100050, China; ^2^China National Clinical Research Centre for Neurological Diseases, Centre of Stroke, Beijing Institute for Brain Disorders, Beijing Key Laboratory of Translational Medicine for Cerebrovascular Disease, Beijing 100050, China; ^3^DDC Clinic-Center for Special Needs Children, Middlefield, OH 44062, USA; ^4^Department of Molecular Biology and Microbiology, Case Western Reserve University School of Medicine, Cleveland, OH 44193, USA; ^5^Department of Structural Biology, University of Pittsburgh School of Medicine, Pittsburgh, PA 45358, USA; ^6^Department of Quantitative Health Sciences, Cleveland Clinic, Cleveland, OH 44193, USA; ^7^Department of Pediatrics, Case Western Reserve University Medical School, Cleveland, OH 44193, USA; ^8^Rainbow Babies & Children's Hospital, Cleveland, OH 44193, USA; ^9^Department of Molecular Cardiology, Cleveland Clinic, Cleveland, OH 44195, USA

## Abstract

*Background*. To investigate whether one or more *SAMHD1* gene mutations are associated with cerebrovascular disease in the general population using a Chinese stroke cohort. *Methods*. Patients with a Chinese Han background (*N* = 300) diagnosed with either cerebral large-artery atherosclerosis (LAA, *n* = 100), cerebral small vessel disease (SVD, *n* = 100), or other stroke-free neurological disorders (control, *n* = 100) were recruited. Genomic DNA from the whole blood of each patient was isolated, and direct sequencing of the *SAMHD1* gene was performed. Both wild type and mutant SAMHD1 proteins identified from the patients were expressed in *E. coli* and purified; then their dNTPase activities and ability to form stable tetramers were analysed *in vitro*. *Results*. Three heterozygous mutations, including two missense mutations c.64C>T (P22S) and c.841G>A (p.E281K) and one splice site mutation c.696+2T>A, were identified in the LAA group with a prevalence of 3%. No mutations were found in the patients with SVD or the controls (*p* = 0.05). The mutant SAMHD1 proteins were functionally impaired in terms of their catalytic activity as a dNTPase and ability to assemble stable tetramers. *Conclusions*. Heterozygous *SAMHD1* gene mutations might cause genetic predispositions that interact with other risk factors, resulting in increased vulnerability to stroke.

## 1. Introduction

Increasing evidence suggests that there is a significant genetic predisposition to cerebrovascular disorders, and these genetic risk factors may account for some portion of the unexplained risk of stroke [1–4]. Identifying these genetic risk factors will not only allow better risk prediction but also provide valuable insights into the mechanism of disease development. The discovery of novel genes associated with stroke may reveal novel pathways involved in stroke pathogenesis, thereby resulting in new targeted treatments and effective prevention [5].

We have recently described a cohort of patients from the Old Order Amish, an inbred population who present a functional loss of the* SAMHD1* (sterile alpha motif and histidine-aspartate (HD) domain-containing protein-1) gene resulting from a homozygous c.1411-2A>G mutation [6]. Although this autosomal recessive condition is heterogeneous, involving multiple systems, the presence of cerebral vasculopathy appears to be a major hallmark of the condition [6, 7]. Similar cerebrovascular findings have also been reported in patients with other* SAMHD1* mutations [8, 9].

The* SAMHD1* gene was originally identified from a human dendritic cell cDNA library as an ortholog of the mouse IFN-*γ*-induced gene* Mg11* [10] and was recently linked to a rare genetic condition, Aicardi-Goutières syndrome [11]. Recent studies have revealed that SAMHD1 is a dGTP-regulated deoxyribonucleoside triphosphate triphosphohydrolase (dNTPase) [12–15], and its tetramerisation is required for biological activity [16, 17]. Due to this structural feature, it has been tempting to speculate that a single mutation in one allele of the* SAMHD1* gene may act in a dominant negative manner with the potential to become pathogenic in humans [18].

Considering the roles of the* SAMHD1* gene in human innate immunity [10], the clinical findings of monogenic linkage studies [11], and its unique structural requirement for catalytic and biological activity [12–18], we hypothesised that the* SAMHD1* gene may be associated with stroke in the general population. In this study, we investigated this hypothesis in a stroke cohort with a completely different ethnic background from that of the original studies linking* SAMHD1* mutations to cerebral vasculopathy.

## 2. Methods

### 2.1. Patients

Patients with a Chinese Han background were recruited from Tiantan Hospital in Beijing, China, from June 2009 to September 2012, either as consecutive outpatients or as inpatients admitted to the Neurology department. Comprehensive clinical evaluations, consisting of a stroke risk assessment and routine hematologic and metabolic assays, including C-reactive protein, erythrocyte sedimentation rate (ESR), and a fasting lipid profile, were performed for each subject. All subjects underwent brain MR imaging with angiography or a CT scan, carotid artery ultrasound, and transcranial Doppler screening. The study was approved by the Ethics Committees/Institutional Review Board of Beijing Tiantan Hospital, Capital Medical University, and written informed consent was obtained from each participant or his/her legal guardian.

Among the 1765 patients with cerebral large-artery atherosclerosis (LAA) and the 428 patients with cerebral small vessel disease (SVD) recruited throughout this investigation, 100 patients from each group were randomly selected to participate in the study. In addition, 100 stroke-free patients were selected as a control group. All 300 subjects were of Chinese Han genetic background. Detailed patient information is summarized in Table 1.

Large-artery atherosclerosis was defined as a stroke caused by atherosclerosis and was categorised as carotid, vertebral, or basilar artery stenosis of more than 50% according to carotid artery ultrasound or MR angiography. A patient with SVD was defined as having one of four imaging features (lacunar infarcts, leukoaraiosis, microbleeds, and dilatation of the perivascular spaces), whereas patients with subcortical lesions of more than 1.5 cm in diameter, a cortical infarct of any size, a potential cardioembolic source, parent artery stenosis, and other large-vessel diseases were excluded. The control group was recruited from stroke-free patients who had another type of neurological disorder, such as epilepsy or Parkinson's disease, but were free of any symptomatic cerebrovascular disease, such as transient ischemic attack. Asymptomatic cerebrovascular diseases were also ruled out in control individuals according to MR imaging or CT scans.

### 2.2. DNA Sequencing and Mutation Identification

Genomic DNA from whole blood collected from each patient was isolated as previously described [19]. PCR primers were designed to amplify each of the 16 protein-coding exons of the* SAMHD1* gene and their flanking intronic sequences. Direct sequencing of the PCR products was performed using an ABI 3130XL sequencer, and sequencing files were analysed using PolyPhred software. Sample sequences were compared with the reference sequences from GenBank to identify sequence variants. GenBank accession numbers NM_015474.3 and NP_056289.2 were used as the* SAMHD1* cDNA and protein sequence references.

### 2.3. Recombinant SAMHD1 Proteins and Activity Assays

Wild type and mutant SAMHD1 proteins bearing missense mutations identified in patients from this cohort were expressed in* E. coli* and purified to near homogeneity as described previously [16, 20]. The effect of mutations on SAMHD1 function was assessed using two assays: (i) dNTPase activity* in vitro* and (ii) the ability to assemble stable tetramers, the functional form of the enzyme [16, 17]. The dNTPase assays were performed in 10 mM Tris-HCl, pH 7.5, 50 mM NaCl, 5 mM MgCl_2_, and 100 nM recombinant SAMHD1 in the presence of the indicated concentrations of dGTP at 25°C. The dG nucleoside products in reaction aliquots were collected at various time points and then quantified by HPLC as described previously [16]. To assess the tetramerisation ability of SAMHD1, 100 *μ*L aliquots of SAMHD1 (250 nM) were mixed with dGTP at the indicated concentrations (0 to 200 *μ*M), injected into the analytical Superdex200 column (24 mL), equilibrated with Tris-HCl, pH 7.8, 50 mM NaCl, 5% glycerol, and 0.02% sodium azide, and separated at a flow rate of 0.8 mL/min [16]. The elution profiles were recorded by monitoring fluorescence traces with an excitation wavelength of 282 nm and an emission wavelength of 313 nm. The areas of the peaks corresponding to the SAMHD1 tetramer (ordinate) were plotted as a function of dGTP concentrations (abscissa).

### 2.4. Statistical Analysis

Age was summarised using means and standard deviations, and gender was summarised using frequencies for patient groups. The Fisher exact test was used to compare the* SAMHD1* mutation frequencies between the groups. A two-sided *p* value less than 0.05 was considered statistically significant. All analyses were conducted using SAS 9.2 (Cary, NC).

## 3. Results

**Figure 1:**
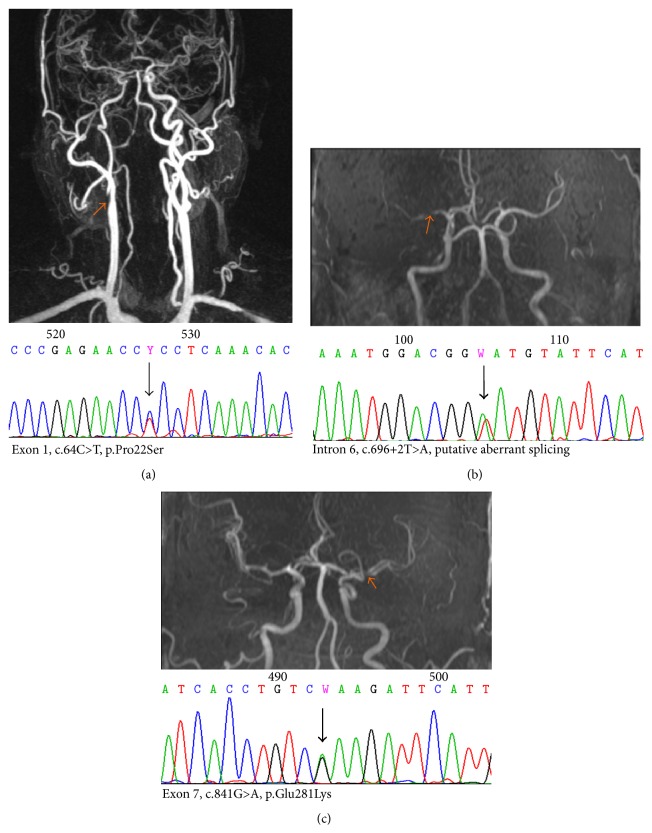
Radiologic findings of the cerebral large arteries in 3 patients with* SAMHD1* gene mutations. In each panel (a–c) the abnormal neuroimaging findings (upper) and sequence electropherogram with the identified* SAMHD1* mutation (lower) are shown. The imaging examination was performed with either contrast-enhanced magnetic resonance angiography (a) or magnetic resonance angiography (b and c). Orange-coloured arrows indicate stenoses of the large arteries and black arrows show the mutations of the* SAMHD1* gene.

**Figure 2:**
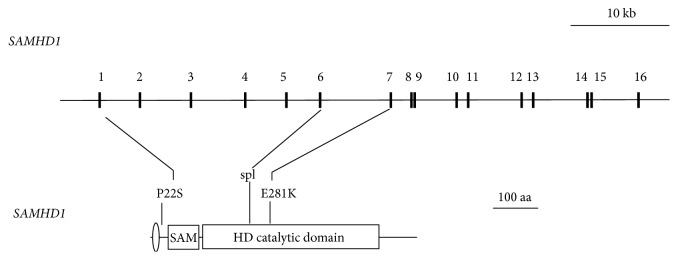
*SAMHD1* mutations in LAA patients. Schematic representation of the 16-exon* SAMHD1* gene (upper panel) and its 626-amino-acid gene product (lower panel). Sixteen exons encode a 626-amino-acid protein that comprises two structural domains. The SAM (sterile alpha motif) and HD (histidine-aspartate) domains are connected by a flexible linker. An oval indicates the location of a nuclear localisation signal. Locations of the missense (P22S, Q281K) and exon 6 splice donor (spl) mutations found in LAA patients are indicated.

Genomic DNA sequencing of the* SAMHD1* gene in all study subjects revealed numerous sequence variants in all three groups (see Supplemental Table e-1 available online at http://dx.doi.org/10.1155/2015/739586). However, of all detected variants, only three variants identified in the LAA group were predicted to affect the protein (Table 2, Figure 1). In contrast, none of the other variants identified in the SVD and control groups caused a change in the SAMHD1 amino acid sequence. The three mutations identified in the LAA group included two missense mutations, c.64C>T (P22S) and c.841G>A (p.E281K), and one splice site mutation c.696+2T>A in intron 6, leading to a putative aberrant splicing event. As shown in Figure 2, the two missense mutations, P22S and E281K, caused amino acid substitutions located proximally to the conserved SAM domain and in the catalytic core of the enzyme, respectively.

The calculated prevalence of* SAMHD1* mutations in the patients of the LAA group was 3%, compared to none in the primary SVD and control groups (*p* = 0.05). The three patients identified with* SAMHD1* mutations in the LAA group were all males. Stroke risk factors, such as hypertension, hyperlipidaemia, hyperhomocysteinaemia, and tobacco and alcohol use, were also identified in all three patients (Table 2).

None of the three variants has been reported previously or is present in the 1000 Genomes Project database (http://www.1000genomes.org/). In the ExAC database (http://exac.broadinstitute.org/), the P22S and E281K missense mutations have only been observed in a frequency of 0.0008% and 0.002%, respectively.* In silico* prediction programs predicted the P22S variant to be deleterious (SIFT) or probably damaging (PolyPhen-2), whereas the E281K was predicted as benign variant by both programs.

**Figure 3:**
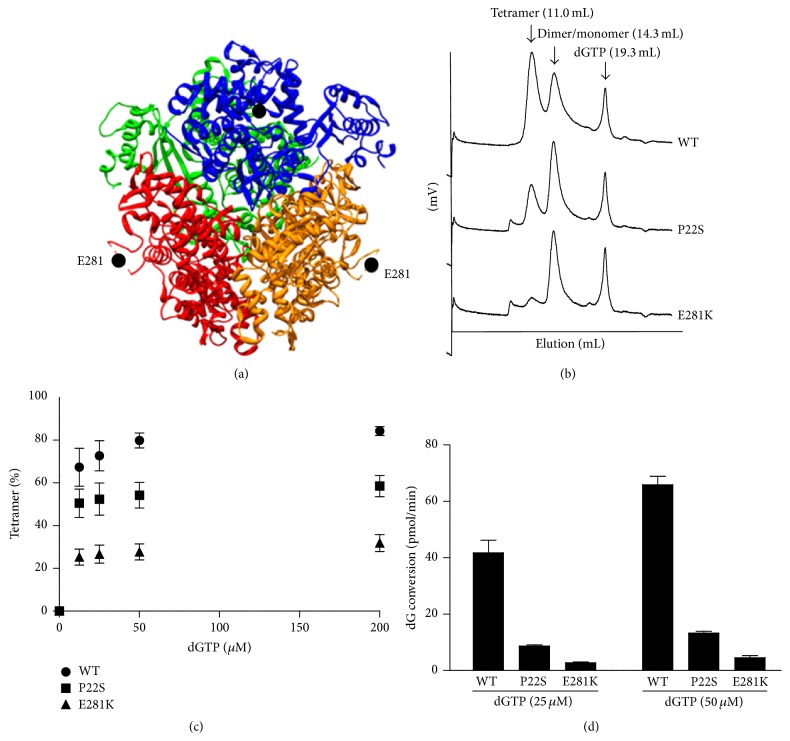
*SAMHD1* mutations from individuals with stroke diminish SAMHD1 tetramerisation and dNTP hydrolase activity. (a) The crystal structure of the tetrameric SAMHD1 catalytic core (residues 113–626; PDB ID, 4BZB) is shown with the position of residue E281 (•) indicated. Residues 278–283 (SPVEDS) were not resolved [17]. Each subunit is rendered with a different colour. (b) SAMHD1 stroke patient mutations interfere with SAMHD1 tetramerisation. Wild type and mutant recombinant SAMHD1 proteins (250 nM) were preincubated with dGTP (25 *μ*M), and the mixtures were separated on an analytical gel filtration column. The peaks corresponding to SAMHD1 tetramers, dimers/monomers, and dGTP are indicated. (c) The extent of SAMHD1 tetramerisation was determined with various concentrations of dGTP (0 to 200 *μ*M) as described in (b). (d) dGTP-dependent dGTPase activity of recombinant SAMHD1. The standard error from triplicate samples is shown.

The association of two missense mutations (P22S and E281K) with stroke suggested that they may have a deleterious effect on SAMHD1-folding and/or function. As a first step to assess this possibility, we examined the recently solved crystal structure of the dGTP-bound SAMHD1 catalytic core (residues 113–626) [17]. The E281 residue is located in the SAMHD1 catalytic core in a loop that appears to be disordered in the structure (Figure 3(a)). The P22 residue resides in the N-terminus, proximal to the SAM domain, and is not present in the structure. Hence, while revealing that both stroke-associated SAMHD1 variant mutations were likely located in unstructured regions, which are known to be frequently involved in interactions with ligands, this information unfortunately failed to provide any direct clues regarding the possible impact of mutations on SAMHD1 structure and function.

We next investigated the biochemical activities of the two substitution variants in the context of full-length SAMHD1 proteins. Biochemical and structural characterisation of SAMHD1 established that the biologically active form is a tetramer induced by dGTP binding at allosteric regulatory sites of the enzyme [16, 17]. Therefore, to assess the possible adverse effects of these mutations on SAMHD1 function, the propensity of SAMHD1 to undergo dGTP-ligand-induced tetramerisation was determined over a wide range of dGTP concentrations. As shown in Figures 3(b) and 3(c), wild type SAMHD1 and both mutants showed increased tetramer content with increasing dGTP concentrations. However, both the P22S and E281K substitutions led to a dramatic reduction in ligand-dependent tetramerisation compared to wild type SAMHD1, even in the presence of the highest dGTP concentration, which indicates that these variants are functionally defective. Thus, we determined the dNTPase activities of both mutants and wild type SAMHD1 as a control (Figure 3(d)). The catalytic activity of the E281K mutant was severely diminished, as predicted by the results of tetramerisation assays (Figures 3(b) and 3(c)). The P22S mutant also showed greatly reduced catalytic activity. Taken together, the results of these functional analyses indicate that both mutations exert a negative effect on the biological functions of SAMHD1.

## 4. Discussion

SAMHD1 was originally identified in a human dendritic cell cDNA library as an ortholog of the mouse IFN-*γ*-induced gene Mg11 and was previously called dendritic cell derived IFN-*γ*-induced protein (DCIP) [10]. High expression levels of the protein in human macrophage and dendritic cells suggested a role in the innate immune response [10, 21, 22]. A previous linkage study of* SAMHD1* with Aicardi-Goutières syndrome associated the gene with a human disease for the first time [11]. Recent studies of* SAMHD1* during HIV infection have significantly increased our understanding of its important role in restricting viral infections [12, 13, 23], as well as its biochemical functions [14–18]. The present study revealed that* SAMHD1* gene mutations are associated with LAA in a Chinese stroke cohort.

In this study, heterozygous mutations have been identified in three patients with LAA, representing 3% of the patients in the group, whereas no mutations were found among 200 patients with SVD or stroke-free controls. We have previously described an autosomal recessive condition in the Old Order Amish population in which a functional loss of* SAMHD1* occurs due to a homozygous c.1411-2A>G mutation [6], with cerebral vasculopathy and an early onset of stroke being major hallmarks. Hence, we have proposed “SAMS (an acronym of cerebrovascular stenosis, aneurysm, moyamoya, and stroke) association” as the name of the disease [7]. The SAMS association seems to affect both large and small cerebral vessels, although cerebral vasculopathy is more predominant in large vessels [6]. In this study, we found that the heterozygous mutations in* SAMHD1* were only associated with LAA.

The phenotype of the patients with heterozygous* SAMHD1* gene mutations appeared less severe than that of SAMS-associated patients. The three individuals with* SAMHD1* mutations from the LAA group showed no signs of other system involvements, unlike the patients with SAMS association [6]. Therefore, based on the course and severity of the disease, one might speculate that the patients with heterozygous mutations manifest a mild type of SAMS association.

However, it should be noted that, along with* SAMHD1* mutations, multiple stroke risk factors, such as hypertension, hyperlipidaemia, hyperhomocysteinaemia, and alcohol and tobacco use, were also identified in all three patients. Thus, we suggest that* SAMHD1* mutations might create a genetic predisposition for stroke that leads to an increased vulnerability to stroke in those patients by interacting with other risk factors.

The exact mechanism of how the* SAMHD1* mutations serve as a genetic predisposition for stroke remains unclear. However, our functional studies indicate that the missense mutations c.64C>T (P22S) and c.841G>A (E281K) identified in the stroke patients impair the function of the SAMHD1 protein (Figures 3(b)–3(d)). It is interesting to note that residue 281 was not resolved in the SAMHD1 dimer structure, an inactive conformer of the enzyme, nor the tetramer, the catalytically active form [16, 17]. In both crystal structures, the loop containing residue 281 resides at the surface of the protein, away from both the ligand-binding allosteric site and the catalytic site. However, the E281K mutant failed to form a stable ligand-bound tetramer and showed a concomitant reduction in its catalytic activity. Thus, it is tempting to speculate that the unresolved region, including residue 281, may interact with a region of SAMHD1 other than the catalytic core to stabilise the tetramer. Consistent with this notion, the P22S mutant located at the N-terminus exerted a similar effect, although the difference was less significant. The other mutation, c.696+2T>A, was predicted to produce a SAMHD1 protein with severely truncated catalytic domains, precluding it from being an active dNTPase. Increasing evidence indicates that SAMHD1 may act as an immunomodulator that plays a protective role by preventing the self-activation of innate immunity [24, 25]. Our reported findings here suggest that impaired functions of this protein might result in an unremittingly proinflammatory status in the affected individuals, thus directly or indirectly initiating progression of the pathological process of LAA. Several lines of evidence implicate SAMHD1 in immune function, as it is upregulated in response to viral infections and is thought to play a role in mediating TNF-*α* proinflammatory responses [22, 26, 27]. However, TNF-*α* is significantly associated with large-artery atherosclerosis [28], suggesting that SAMHD1 may initiate the progression of LAA via the TNF-*α* pathway. Indeed, abnormal laboratory findings, including elevated ESR, immunoglobulin G, neopterin, and TNF-*α*, have been found in patients with the homozygous mutation [6], whereas moderately increased levels of cytokines have been observed in heterozygous carriers of the c.1411-2A>G mutation (unpublished data).

The vast majority of strokes are increasingly recognized as polygenic events. Although monogenic causes of stroke are rare, identification of these genes and mutations is important to provide critical information for the diagnosis, treatment, and prognosis of affected individuals. Furthermore, identifying novel gene variants associated with stroke may reveal novel pathways involved in stroke pathogenesis and thus result in new targeted treatments and more effective prevention of stroke. In this study, although the incidence of* SAMHD1* mutations in LAA patients was only 3%, this prevalence is noteworthy in light of the complexity of the multiple genetic and environmental risk factors that influence the disease. Because the study was intentionally performed in a completely different population from that of the original studies linking* SAMHD1* mutations and cerebral vasculopathy, the implication may be more prominent. Further studies involving additional patient populations and exploring the mechanism underlying the effect of* SAMHD1* mutations on the development of LAA in the general population will be valuable, not only for patients who are directly affected but also for stroke patients in general.

## Supplementary Material

SAMHD1 sequencing and mutation identification
SAMHD1 whole gene sequencing was performed for each patient from LAA, SVD, and control groups recruited for this study. Sequence variants identified from all protein coding exons, exon-intron boundaries, and untranslated regions (5'- and 3'-UTRs) are collected and listed in Table e-1. GenBank accession numbers NM_015474.3 and NP_056289.2 were used as the SAMHD1 cDNA and protein sequence references.

## Figures and Tables

**Table 1:** Age and gender distributions among the patients.

	LAA	SVD	Control
	(*n* = 100)	(*n* = 100)	(*n* = 100)
Age (years)	59.3 ± 9.5	59.8 ± 9.1	62.9 ± 6.3
Gender (M/F)	76/24	55/45	50/50

LAA = cerebral large-artery atherosclerosis; SVD = cerebral small vessel disease.

Control = stroke-free control.

The ages are expressed as the means ± SD, with *N* in parentheses indicating the total number of patients in each group.

**Table 2:** Additional clinical features in patients with *SAMHD1* mutations.

	Gender	Age (years)	Stroke risk factors	Mutations identified
Case 1	M	58	Hypertension	Exon 1 c.64C>T (p.Pro22Ser)
			Hyperlipidaemia	
			Hyperhomocysteinaemia	
			Smoking	
			Drinking	

Case 2	M	39	Hyperlipidaemia	Intron 6 c.696+2T>A (putative aberrant splicing)
			Smoking	
			Drinking	

Case 3	M	56	Hypertension	Exon 7 c.841G>A (p.Glu281Lys)
			Hyperlipidaemia	
			Smoking	
			Drinking	
