# Supplementary material for: SAMHD1 Gene Mutations Are Associated with Cerebral Large-Artery Atherosclerosis
Source: Biomed Res Int. 2015 Oct 4;2015:739586. doi: 10.1155/2015/739586 (PMC4609382; doi:10.1155/2015/739586)
Supplement: Supplementary file 1 — SAMHD1 sequencing and mutation identification SAMHD1 whole gene sequencing was performed for each patient from LAA, SVD, and control groups recruited for this study. Sequence variants identified from all protein coding exons, exon-intron boundaries, and untranslated regions (5'- and 3'-UTRs) are collected and listed in Table e-1. GenBank accession numbers NM_015474.3 and NP_056289.2 were used as the SAMHD1 cDNA and protein sequence references. [file 739586.f1.doc]

**Table e-1. *SAMHD1* sequence variants identified in the three groups**

| **Phenotype** | **Exon/Intron** | **DNA change** | **Protein change** |
| --- | --- | --- | --- |
| LAA | 5'-UTR | c.-110T>C |  |
|  | 5'-UTR | c.-32C>G |  |
|  | Exon 1 | c.64C>T | p.Pro22Ser |
|  | Intron 2 | c.276-105C>A |  |
|  | Intron 3 | c.348+159A>C |  |
|  | Intron 4 | c.510-74T>G |  |
|  | Intron 6 | c.696+2T>A | Putative splice site |
|  | Intron 6 | c.697-263_697-260del |  |
|  | Exon 7 | c.841G>A | p.Glu281Lys |
|  | Intron 12 | c.1411-38A>T |  |
|  | Intron 13 | c.1503+114_1503+115ins |  |
|  | Intron 15 | c.1746+213A>G |  |
|  | 5'-flanking | c.-345G>A |  |
| SVD | 5'-flanking | c.-299C>T |  |
|  | 5'-UTR | c.-150C>G |  |
|  | 5'-UTR | c.-110T>C |  |
|  | Intron 2 | c.276-105C>A |  |
|  | Intron 3 | c.348+159A>C |  |
|  | Intron 3 | c.349-286C>T |  |
|  | Intron 4 | c.510-103G>C |  |
|  | Intron 4 | c.510-74T>G |  |
|  | Intron 5 | c.625+47T>G |  |
|  | Intron 6 | c.696+27A>G |  |
|  | Intron 6 | c.697-263_697-260del |  |
|  | Intron 10 | c.1154+126G>A |  |
|  | Intron 10 | c.1155-122G>C |  |
|  | Intron 12 | c.1410+71C>T |  |
|  | Intron 12 | c.1411-31A>G |  |
|  | Intron 13 | c.1503+114_1503+115ins |  |
|  | Exon 16 | c.1791A>G | p.(=) |
|  | 3'-UTR | c.*54G>C |  |
|  | 5'-UTR | c.-110T>C |  |
| Control | 5'-UTR | c.-32C>G |  |
|  | Intron 2 | c.276-193delC |  |
|  | Intron 2 | c.276-105C>A |  |
|  | Intron 3 | c.348+159A>C |  |
|  | Intron 4 | c.510-74T>G |  |
|  | Intron 6 | c.697-263_697-260del |  |
|  | Intron 12 | c.1411-18G>A |  |
|  | Intron 13 | c.1503+114_1503+115ins |  |
|  |  |  |  |
